# The effect of pasture allowance and amount of concentrate offered to grazing dairy cows on milk production, dry matter intake, and grazing behavior

**DOI:** 10.3168/jdsc.2025-0952

**Published:** 2026-03-27

**Authors:** Meaghan L. Douglas, Joanna W. Heard, William J. Wales, Khageswor Giri, Greg L. Morris, Monique J. Berkhout, Joseph L. Jacobs, Joanna E. Newton, Marlie M. Wright

**Affiliations:** 1 Agriculture Victoria Research, Department of Energy, Environment and Climate Action, Ellinbank, VIC 3821, Australia; 2 Agriculture Victoria Research, Department of Energy, Environment and Climate Action, Hamilton, VIC 3300, Australia; 3 AgriBio, Agriculture Victoria Research, Department of Energy, Environment and Climate Action, Bundoora, VIC 3086, Australia; 4 School of Applied Systems Biology, La Trobe University, Bundoora, VIC 3083, Australia

## Abstract

The dairy industry in temperate regions of the world is predominantly pasture-based, with concentrates often fed twice daily in the parlor during milking. Intake of pasture is influenced by the amount of pasture allocated, affecting postgrazing residuals and pasture utilization. This experiment investigated the milk production, DMI, and grazing behavior of dairy cows offered different pasture allowances (PA) and different amounts of concentrate. Thirty-six Holstein-Friesian dairy cows were used in the 25-d experiment, which included two 6-d measurement periods. Three cows were randomly assigned to each of the 12 treatment combinations using a completely randomized design, balanced for DIM and both BW and milk production based on data from the 7 d before the beginning of the experiment. The treatment combinations consisted of offering cows one of 4 PA (20, 35, 50, or 65 kg DM/cow per day; to ground level) and one of 3 concentrate amounts (4, 6, or 8 kg DM/cow per day). Cows swapped PA amounts for each measurement period, but the concentrate amount offered to each cow was unchanged. Average daily milk yield was greater for cows offered PA at and above 35 kg DM/cow per day, as was pasture DMI. Cows offered 6 or 8 kg DM of concentrate per day had a greater average milk yield compared with cows offered 4 kg DM of concentrate per day, but lower pasture DMI. Total daily eating or ruminating time were not significantly affected by either PA or the amount of concentrate fed, but cows offered 4 kg DM of concentrate per day had a greater number of graze bites per day. Finding optimal PA and concentrate amounts to offer is key to adequately feeding cows and managing pasture growth and utilization on pasture-based dairy farms. Because there is considerable variation in geographic region and management strategies of pasture-based farms, which may influence on-farm feed allocation, these data provide information for farmers on what potential benefits they could achieve.

## Highlights

• Milk yield was highest when cows were offered PA at and above 35 kg DM/cow per day.
• Milk yield was highest when cows were offered concentrate at 6 or 8 kg DM/cow per day.
• Offering a higher PA (above 35 kg DM/cow per day) resulted in increased pasture DMI.
• Postgrazing pasture mass increased as PA increased.
• Neither PA nor concentrate amount influenced daily eating or ruminating time.

## Graphical Abstract

This experiment investigated the effects of offering different pasture allowances (PA) and concentrate amounts on milk production, individual cow dry matter intake (DMI), and grazing behavior of dairy cows. An understanding of how the amount of feed offered influences cow production and intake will positively influence how it is allocated to ensure good pasture management. Thirty-six Holstein-Friesian cows were assigned to one of 4 PA and one of 3 concentrate amounts, and monitored for 25 days, including two 6-day measurement periods. A PA offered at and above 35 kg dry matter (DM)/cow per day increased milk production and pasture DMI. However, offering PA above 35 kg DM/cow per day resulted in underutilization of pasture. Higher milk yields were also observed when cows were offered 6 or 8 kg DM of concentrate per day, but these cows had lower pasture DMI compared with cows offered 4 kg DM of concentrate per day. To remain profitable, it is vital that managers of pasture-based dairy farms understand the interactions between PA, pasture DMI, pasture utilization, and substitution when supplements are fed.

**Figure:**
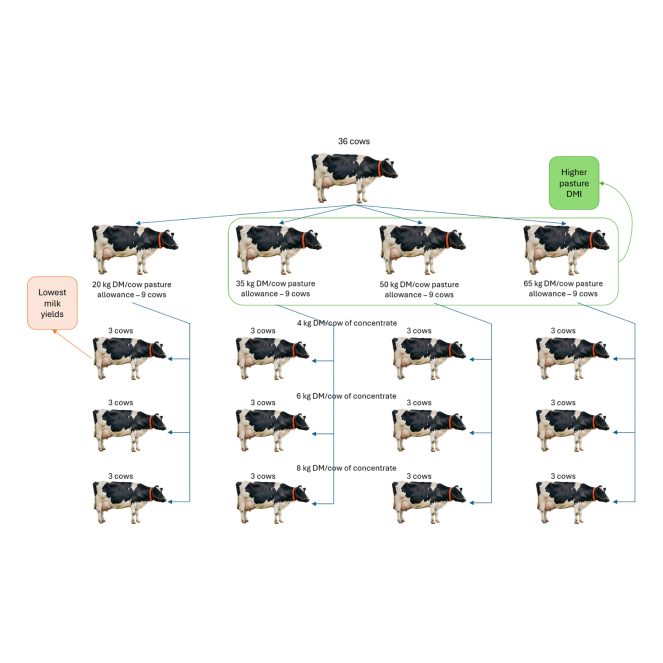

Milk production in countries such as Australia, Ireland, and New Zealand is mostly pasture-based (Doyle and Stockdale, 2011). Pasture allowance (**PA**) is the primary factor influencing intake and milk production. Australian research showed that pasture DMI increased as PA increased from 20 to 70 kg DM/cow per day, regardless of pasture mass (Wales et al., 1999). Although increasing PA increases pasture DMI (Peyraud et al., 1996; Pérez-Prieto and Delagarde, 2012), studies have reported that this reduces pasture utilization; for example, pasture utilization was lower for cows offered a higher PA, regardless of herbage mass (1,600 vs. 2,400 kg DM/ha; Curran et al., 2010), for pastures measured to grazing height (4 cm). Grazing behavior has also been reported to be influenced by PA, and when PA is high, cows spend less time eating and more time ruminating (Zanine et al., 2019; Alvarez-Hess et al., 2021). Increasing PA also increases milk production by providing a higher intake of DM and nutrients (Bargo et al., 2002; Kennedy et al., 2007). Therefore, there are clear benefits for an increased PA for milk production, but likely a trade-off in terms of pasture utilization.

Although pasture forms the basal part of the diet for dairy cows in temperate regions of the world, the cows are often supplemented with concentrates fed in the parlor twice daily during milking (Kellaway and Harrington, 2004), as pasture alone cannot sustain high milk yields (Kolver and Muller, 1998). Pasture utilization is reduced when the amount of concentrate offered increases, resulting in substitution, which influences postgrazing residuals (Stockdale, 2000). Douglas et al. (M. L. Douglas, J. W. Heard, K. Giri, G. L. Morris, M. J. Berkhout, W. J. Wales, J. L. Jacobs, J. E. Newton, and M. M. Wright; unpublished) found that cows offered 7 kg DM of concentrate per day consumed less pasture than cows offered either 3 or 5 kg DM, but total diet DMI did not differ between cows fed any of the 3 concentrate amounts when pregrazing pasture height was 14 cm (average pregrazing pasture mass of 3,670 kg DM/ha, measured to ground level). Profitable milk production requires sound grazing management balancing both the amount of pasture allocated and the amount of concentrate fed to ensure efficient utilization of available pasture, as it is the cheapest feed source.

This experiment aimed to investigate the impacts of offering pasture to grazing dairy cows at one of 4 PA (to ground level) and offering one of 3 amounts of concentrate on milk production, DMI, and grazing behavior. The hypotheses tested here were that (1) increasing PA would result in higher pasture DMI and higher postgrazing pasture mass but lower pasture utilization; (2) increasing PA and the amount of concentrate offered would increase milk production; and (3) total daily eating time would be greater for cows offered a higher PA, but not a higher concentrate amount. Additionally, although PA over 40 kg DM/cow per day are less common in Australian dairy systems, high PA were included in this experiment to provide additional data that could be used to validate DMI prediction equations (J. W. Heard, K. Giri, M. L. Douglas, and M. Wright, unpublished).

The experiment was conducted in September 2023 (spring) at the Agriculture Victoria Research Ellinbank SmartFarm (Victoria, Australia; 38°14′S, 145°56′E) with approval from the Department of Energy, Environment and Climate Action (DEECA) Agricultural Research and Extension Animal Ethics Committee (AEC approval number 2023-10).

Thirty-six spring-calving Holstein-Friesian cows were offered one of 4 PA (20, 35, 50, or 65 kg DM/cow per day; to ground level) and one of 3 concentrate amounts (4, 6, or 8 kg DM/cow per day). Three cows were randomly allocated to each of the 12 treatments using a completely randomized design. Each treatment was balanced for cow age and DIM, as well as 7-d average BW and 7-d average milk production (determined in the 7 d before commencement of the experiment). Cows were in at least their second lactation, between 3 and 10 yr of age, and were in early lactation at an average of 37 DIM (range 23 to 63 DIM) at the beginning of the experiment. The average previous 305-d milk yield of these cows was 8,256 kg.

The experiment was conducted over 25 d. During the adaptation period (the first 4 d) cows were adapted to their allocated PA and amount of concentrate. From the beginning of the 6-d dosing period (commencing on d 5), cows began to graze in plots in their allocated treatment groups, and the n-alkane technique was employed to estimate pasture DMI in individual cows. The 12 treatment groups were grazed adjacent to each other in the same paddock. The experiment included two 6-d measurement periods (d 11 to 16, and d 19 to 24) separated by a 2-d wash-out period during which the cows on the 20 kg DM/cow per day PA swapped to the 35 kg DM/cow per day PA, and vice versa, and cows on the 50 kg DM/cow per day PA swapped to the 65 kg DM/cow per day PA, and vice versa, similar to a crossover design. The concentrate allocations were unchanged, with n-alkane dosing continuing during the wash-out period.

Daily pasture allocation (measured to ground level) was split into 2 equal halves, with a fresh strip of pasture allocated after each milking. Back-fencing was used to prevent cows from re-grazing the previous day's pasture. Pasture allowance was measured by calibrating a rising plate meter (Ellinbank Plate Meter; Earle and McGowan, 1979) using quadrat cuts to construct regression equations that related kilograms of DM per hectare to plate meter readings of compressed pasture height. Estimates of pasture mass were used to determine the area of the paddock required to achieve the correct PA for each treatment for the duration of the experiment.

In addition to their respective PA, cows were offered either 4, 6, or 8 kg DM of concentrate per day. The concentrate mix (Reid Stockfeeds, Trafalgar, VIC, Australia) consisted of (% of DM offered) 12.5% crushed wheat, 60.0% crushed corn, 20.0% canola meal, and 7.5% minerals (mineral mix consisted of AMS Go Cow 200 [Animal Mineral Solutions, Shepparton, VIC, Australia], magnesium, limestone, salt, and sodium bicarbonate), and was fed twice daily in equal portions in the parlor during milking. Concentrate intake was calculated as the concentrate offered minus the concentrate refused.

The milk yield of every cow was recorded twice daily according to normal farm practice using a DeLaval Delpro milk metering system (DeLaval International, Tumba, Sweden), and milk composition was measured 3 times (p.m. and a.m.; 6 milkings in total) during each of the two 6-d measurement periods using the methodology described by Douglas et al. (2021). Energy-corrected milk yield standardized to 4.0% fat and 3.3% protein was calculated using the following formula (Tyrrell and Reid, 1965):

ECM (kg/cow per day) = milk yield (kg) × (376 × fat% + 209 × protein% + 948)/3,138.

The BW of each cow was measured twice daily by walking cows over electronic scales (Automatic Weight System AWS100, DeLaval, Tumba, Sweden) after each milking.

Individual cow DMI of pasture was estimated using the n-alkane technique developed by Mayes et al. (1986). All cows were dosed twice daily with gelatin capsules containing a known concentrate of a synthetic n-alkane for 20 d, and fecal samples were collected from each cow twice daily during each of the two 6-d measurement periods using the methodology described by Wright et al. (2025). Representative samples of the pregrazing pasture offered were collected daily for each of the 12 treatments, as were representative samples of the concentrate offered to the cows. These samples were processed as per Wright et al. (2025).

The concentrations of n-alkanes were analyzed at a commercial laboratory (AgriBio, Bundoora, Australia) by GC using the method described by Dove and Mayes (2006) and modified by Liu et al. (2022). The average daily individual pasture DMI (kg DM/cow per day) of each cow was estimated from the concentrations of the n-alkanes in feces, the concentrate mix and pasture, and the daily dose rate of C_32_. Average daily pasture intake (kg DM/cow per day) was estimated using the following equation (Dove and Mayes 1991):

$$\mathrm{intake}=\frac{\left(\frac{F_{i}}{F_{j\;}}\times \left(D_{j\;}+I_{c}\times C_{j}\right)-I_{c}\times C_{i}\right)}{\left(H_{i}-\frac{F_{i}}{F_{j}}\times H_{j}\right)},$$

where *F_i_* and *H_i_* represent the fecal and herbage n-alkane concentrations of the C_33_ alkane, respectively; *F_j_* and *H_j_* represent the fecal and herbage n-alkane concentrations of the C_32_ alkane, respectively; *D_j_* represents the daily dose rate of the C_32_ alkane; *I_c_* represents the known intake of the concentrate mix; *C_i_* represents the n-alkane concentrations of the C_33_ alkane in the concentrate mix; and *C_j_* represents the n-alkane concentrations of the C_32_ alkane in the concentrate mix.

Pasture samples for nutritive analysis were collected before and after grazing for each of the 12 treatments during each day of the two 6-d measurement periods, and processed following the methodology of Douglas et al. (2025). Samples were sent for analysis at a commercial laboratory (Dairy One Forage Laboratory, Ithaca, NY) for nutritive characteristics (CP, NDF, ADF, starch, NFC, lignin, ash, and calculated ME) by wet chemistry (AOAC International, 2000; Dairy One, 2020). A sample of the concentrate mix in each measurement period was also collected and analyzed by wet chemistry at a commercial laboratory (Dairy One Forage Laboratory, Ithaca, NY).

Grazing behavior was estimated for all cows during each of the two 6-d measurement periods using RumiWatch noseband sensors (Itin and Hoch GmbH, Liestal, Switzerland). The noseband sensors were used to measure eating, ruminating, and idling times, as well as the number of bites and chews. Data were analyzed using RumiWatch Manager 2 software (version 2.2.0.0) and RumiWatch Converter software (version 0.7.4.13; Itin and Hoch GmbH). Data were converted into hourly summaries and then summed for each 24-h period (0600 h to 0600 h the following day) before analysis.

Daily milk yield, milk composition, and pasture intake data were averaged across each measurement period to obtain a mean value for each cow within each period. Feeding behavior data and activity durations were summed over each 24-h interval (0600 h to 0600 h) and then averaged across each measurement period for each cow. These cow-level mean values were analyzed using an analysis of covariance (**ANCOVA**) model. The ANCOVA model included the main effects of PA and concentrate level, and their interaction, as the treatment structure, with effects of cows and period within cow as the blocking structure. Pre-experimental measurements (age, DIM, BW, and average milk yield) were included as covariates in the ANCOVA model.

Model assumptions were checked by examining histograms of residuals and plots of residuals versus fitted values for normality and homogeneity of variance. Treatment effects were considered significant at *P* < 0.05. Pairwise comparisons among treatment means were performed using Fisher's protected LSD test, with different superscript letters used to indicate significant differences. All statistical analyses were conducted using GenStat (version 23, VSN International Ltd., Hemel Hempstead, UK).

Interaction effects between PA and concentrate level were not significant for any of the variables measured, including daily milk yield, milk composition, BW, pasture intake, and grazing behavior. Therefore, the results are presented and discussed in terms of main effects only.

Pasture DMI was greatest (*P* < 0.001; Table 1) for cows offered a PA at and above 35 kg DM/cow per day compared with cows offered a PA of 20 kg DM/cow per day. Research undertaken in Australia has shown increased pasture DMI when PA was increased from 15 to 40 kg DM/cow per day, even when 12 kg DM of a partial mixed ration was also offered to cows (Auldist et al., 2016; 2017). In our experiment, an increase in pasture DMI was seen when PA increased from 20 to 35 kg DM/cow per day, but there was no significant increase with further increases in PA. Although more pasture was available for these cows to eat, there is a limit to the amount of feed that cows can consume, even in early lactation.

**Table 1:** Mean, standard error of the difference (SED), and *P*-values of the main effects, including milk yield (plus yields of milk fat, protein and lactose), ECM, milk composition, BW, and DMI (concentrate, pasture, and total), for cows offered one of 4 pasture allowances, and one of 3 concentrate amounts, averaged over the two 6-d measurement periods

Variable	Pasture allowance (kg DM/cow per day)				Concentrate amount (kg DM/cow per day)			SED		*P*-value	
	20	35	50	65	4	6	8	Allowance	Concentrate	Allowance	Concentrate
Concentrate intake^1^ (kg DM)	6.0	6.0	5.9	5.9	4.0	6.0	8.0	—	—	—	—
Pasture intake^1^ (kg DM)	19.9^a^	22.8^b^	23.1^b^	24.2^b^	24.2^b^	21.7^a^	21.7^a^	0.82	0.71	<0.001	<0.001
Total intake (kg DM)	25.9^a^	28.8^b^	29.0^b^	30.1^b^	28.2^a^	27.7^a^	29.7^b^	0.81	0.71	<0.001	0.019
Milk yield (kg/cow per day)	36.6^a^	38.3^ab^	38.6^b^	39.3^b^	36.6^a^	38.6^b^	39.4^b^	0.93	0.81	0.034	0.003
ECM (kg/cow per day)	33.2^a^	35.7^b^	36.0^b^	36.6^b^	33.9^a^	35.5^ab^	36.7^b^	1.03	0.90	0.006	0.013
Milk protein (kg/cow per day)	1.10^a^	1.19^b^	1.21^b^	1.23^b^	1.13^a^	1.20^b^	1.21^b^	0.035	0.030	0.002	0.017
Milk protein (%)	3.02	3.11	3.13	3.15	3.09	3.13	3.09	0.073	0.063	0.261	0.710
Milk fat (kg/cow per day)	1.27	1.35	1.35	1.38	1.30	1.33	1.39	0.053	0.046	0.147	0.096
Milk fat (%)	3.47	3.56	3.52	3.52	3.54	3.47	3.55	0.143	0.125	0.944	0.791
Milk lactose (kg/cow per day)	1.80^a^	1.89^ab^	1.91^b^	1.94^b^	1.79^a^	1.89^ab^	1.97^b^	0.052	0.046	0.047	<0.001
Milk lactose (%)	4.92	4.93	4.95	4.93	4.90^a^	4.90^a^	5.00^b^	0.041	0.036	0.854	0.008
Bodyweight (kg)	601^a^	612^ab^	619^b^	621^b^	616	609	615	5.5	4.8	0.003	0.277
Total daily eating^2^ time (min/d)	475	468	472	518	509	466	473	30.3	26.4	0.339	0.235
Graze bites (number/d)	26,425	25,328	24,512	26,196	28,714^b^	24,452^a^	23,680^a^	2,006	1,751	0.740	0.012
Eat up^3^ chews (number/d)	5,882	5,484	6,038	7,114	6,130	5,427	6,831	1,253	1,093	0.623	0.439
Eat down^3^ chews (number/d)	29,319	28,182	27,516	29,745	30,956	27,981	27,134	2,177	1,900	0.703	0.113
Ruminating time (min/d)	495	513	470	515	521	480	493	25.9	22.6	0.268	0.212
Ruminating chews (number/d)	30,709	31,815	29,084	32,194	32,123	30,086	30,642	1,790	1,563	0.298	0.413

a,b Within a row, different superscripts indicate significant differences between treatments (*P* < 0.05).

1 Concentrate intake determined by calculating the amount of feed offered minus feed refused; pasture intake determined using the n-alkane technique.

2 Total daily eating time: pasture and concentrate.

3 Eat up and eat down chews: chews made with the cow's head either up or down, respectively.

The pasture DMI measured in this experiment using the n-alkane technique was higher than expected, even at higher PA. Intake of pasture was unlikely to be affected by the nutritive characteristics of the pasture, which averaged 10.5 MJ of ME, 16.2% CP, and 48.7% NDF across all treatments. Other research with pasture of this quality has achieved lower pasture DMI with PA levels similar to the 20 and 35 kg DM/cow per day allowances; cows consumed a maximum of 13.4 kg DM of pasture per day in research by Douglas et al. (2025) on a PA of 28 kg DM/cow per day, whereas cows offered pasture with a lower ME and CP but higher NDF than reported here consumed a maximum of 17.2 kg DM of pasture per day in research by Douglas et al. (2021). The cause of the higher estimated DMI than expected in our experiment is unknown, but could possibly be related to the quality of the pasture and fecal samples collected for analysis. Previous research concluded that although the n-alkane technique was the best technique to use for estimating the pasture intake of individual grazing animals, estimates of intake by the n-alkane technique overestimated pasture intake compared with estimates calculated using the energy requirements of the animal (Smit et al., 2005).

Cows offered 4 kg DM of concentrate per day had the greatest (*P* < 0.001) pasture intake (24.2 kg DM) compared with cows offered either 6 or 8 kg DM of concentrate per day. By design, average concentrate DMI was not different between cows on any of the 4 PA, but the variation in pasture DMI between cows on the different allowances resulted in differences in total DMI (Table 1). Cows offered PA at and above 35 kg DM/cow per day had a greater (*P* = 0.019) total DMI than cows offered 20 kg DM/cow per day.

Allocating pasture to a herd of milking dairy cows requires finding a balance between efficient and profitable milk production and pasture utilization. Pregrazing pasture mass was similar between the 12 PA by concentrate intake treatments. However, the postgrazing mass and the amount of pasture removed in each treatment varied in the PA by concentrate intake interaction (Table 2). Cows offered each of the 4 PA removed an average of 2,020 kg DM/ha (20 kg DM/cow per day), 1,480 kg DM/ha (35 kg DM/cow per day), 1,190 kg DM/ha (50 kg DM/cow per day), and 1,050 kg DM/ha (65 kg DM/cow per day) over each day of the two 6-d measurement periods. Figure 1 shows the average pre- and postgrazing pasture mass of each of the 12 treatments over both measurement periods, with a line indicating the target postgrazing pasture mass of 1,500 kg DM/ha. Cows offered a PA of 20 kg DM/cow per day consumed their allocated pasture close to the preferred residual mass, whereas cows offered 35 kg DM/cow per day and above left a large amount of pasture (Table 2).

**Table 2:** Pre- and postgrazing pasture mass (kg DM/ha), the mass of pasture removed (kg DM/ha) in each of the 12 pasture allowance by concentrate amount interaction treatments, and pasture utilization (%)

Item	Interaction^1^											
	20_4	20_6	20_8	35_4	35_6	35_8	50_4	50_6	50_8	65_4	65_6	65_8
Pregrazing mass	3,910	3,410	3,860	4,000	3,620	3,860	3,590	3,910	3,800	3,900	3,880	3,800
Postgrazing mass	1,770	1,570	1,780	2,360	2,160	2,520	2,490	2,650	2,590	2,670	2,930	2,810
Mass removed	2,140	1,840	2,080	1,640	1,460	1,340	1,100	1,260	1,210	1,230	950	990
Pasture utilization^2^	55	54	54	41	40	35	31	32	32	32	24	26

1 Allowance by concentrate amount treatments (daily pasture allowance_daily concentrate amount).

2 Pasture utilization calculated using the equation by Langworthy et al. (2021).

**Figure 1:**
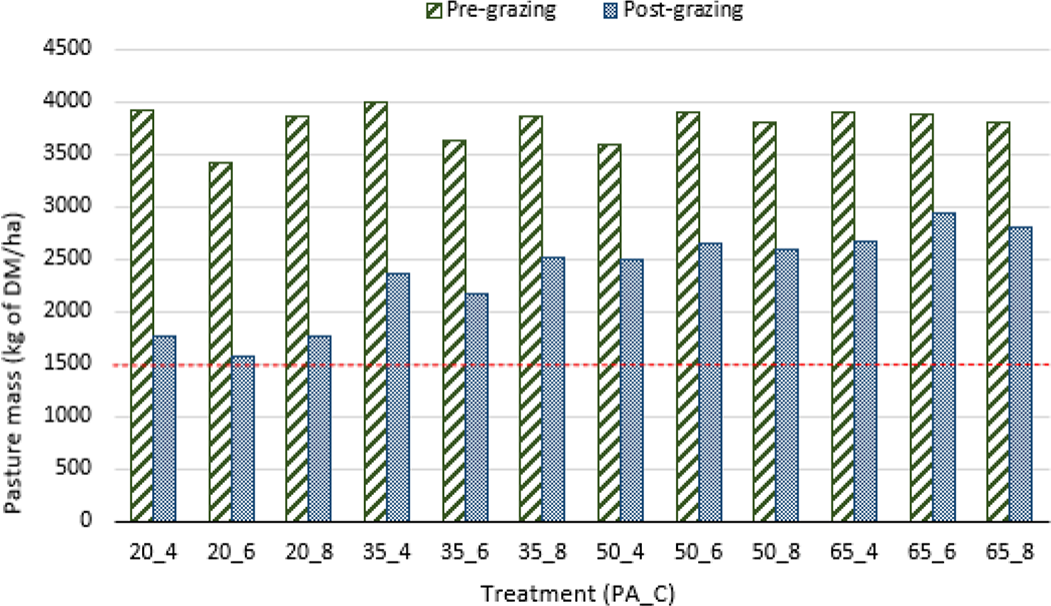
Pre- and postgrazing pasture mass (kg DM/ha) in each of the 12 distinct pasture allowance by concentrate intake amount (PA_C) treatments. The dotted red line shows the target postgrazing residual (1,500 kg DM/ha).

Increasing the amount of pasture available to cows reduces pasture utilization, as shown in our experiment. Pasture utilization averaged 54%, 39%, 32%, and 27% for cows offered PA of 20, 35, 50, and 65 kg DM/cow per day, respectively (Table 2). Auldist et al. (2013) reported pasture utilization ranges of 60% to 66% for cows offered 6 to 12 kg DM/d of wheat when PA was 13.5 to 14.5 kg DM/cow per d, whereas pasture utilization for cows offered 8 kg DM of concentrate per day and a PA of 20 kg DM/d in the current experiment was 54%. When cows do not graze the pasture down to the recommended residual, this can affect successive pasture regrowth and the nutritive value of the pasture. For example, Burggraaf et al. (2018) showed that CP and ME were reduced in summer pasture in successive grazings when a postgrazing residual at or above 1,800 kg DM/ha was achieved. This occurred for all treatments with a PA at and above 35 kg DM/cow per day in our experiment. Therefore, pasture was underutilized at the higher PA. Poole et al. (2019) reported that cows consuming higher amounts of supplementary feed had a lower pasture utilization, resulting in increased postgrazing residuals; these could be improved if the allowance is adjusted to account for this.

It could be postulated that a PA between 20 and 35 kg DM/cow per day should be offered to ensure high levels of pasture DMI with acceptable pasture utilization. In our experiment, pasture utilization increased from an average of 39% to 54% when PA was reduced from 35 kg DM/cow per day to 20 kg DM/cow per day. Additionally, there was statistically no increase in total DMI nor milk production when a PA greater than 35 kg DM/cow per day was offered. Wales et al. (1998) reported an increase in pasture intake as PA increased (from 8.0 to 14.6 kg DM as PA increased from 15 to 40 kg DM/cow per day) with an associated decrease in utilization (54% to 37%). The postgrazing residual was also increased in that experiment, and it was concluded that postgrazing pasture mass was a consequence of grazing behavior. The exact PA offered would differ between farms depending on factors including but not limited to the type and amount of concentrate offered. Feeding pasture at a higher allowance increases substitution when also supplementing with concentrates, where a greater decrease in pasture intake per kilogram of concentrate eaten occurs at higher PA (Grainger and Mathews, 1989). Therefore, pasture utilization will be reduced further if concentrate supplementation is too high, a feature observed in our experiment with higher postgrazing residuals on higher PA when more concentrate (6 or 8 kg DM of concentrate/cow per day) was offered (Figure 1).

Average daily milk and ECM yields were higher for cows offered a PA at and above 35 kg DM/cow per day (*P* < 0.05; Table 1), but we found no difference in yields between the 3 higher allowances. Our results are consistent with those of Wales et al. (1999), who reported an increase in milk production when PA increased, and Bargo et al. (2002) also showed that milk yield increased when PA increased from 27 to 49 kg DM/cow per day. Chilibroste et al. (2012) reported an increased milk response to higher PA up to 30 kg DM/cow per day with first-lactation heifers.

Cows offered a PA of 20 kg DM/cow per day had the lowest milk protein and milk lactose yields (*P* < 0.05), but we found no difference between the treatments for milk fat yield. Previous research has shown a decline in milk production with restricted PA (60% of the control allocation) as well as an 18% decrease in milk solids (milk fat + milk protein) yield (Claffey et al., 2020). Results from our experiment show a 9% decrease in milk solids yield between cows offered a PA of 20 kg DM/cow per day compared with those offered 65 kg DM/cow per day. Differences in PA did not influence concentrations of milk protein, fat or lactose in our experiment, with research by Maher et al. (2003) also reporting no change in milk protein concentration when PA increased.

Daily milk yield and milk protein yield were greater for cows offered either 6 or 8 kg DM of concentrate (average 39.0 kg and 1.21 kg, respectively; *P* < 0.05) than for cows offered 4 kg DM of concentrate/d (36.6 kg and 1.13 kg, respectively; Table 1). Evers et al. (2021) found that pasture availability had no effect on milk production, but increased concentrate allowance resulted in greater milk and milk solids yields. It is well known that milk production increases when cereal grain is fed (Walker et al., 2001; Leddin et al., 2009) but over-feeding can have deleterious effects on rumen pH, which affects DMI and subsequent milk production (Wales and Doyle, 2003; Doyle et al., 2005; Auldist et al., 2013). Auldist et al. (2016) demonstrated an increase in milk production with increasing supplement intake, including when comparing a cereal grain-only diet with a concentrate mix containing a protein supplement. These authors concluded that the mechanisms behind the increase in milk production for cows fed more concentrate are likely to be multifold, and include an increase in the amount of pasture consumed, within the constraints of PA. As discussed earlier, cows offered PA at and above 35 kg DM/cow per day had both greater pasture DMI and milk production compared with cows offered a PA of 20 kg DM/cow per day.

Grazing behavior, such as the daily total eating and ruminating times, were not affected by either PA or amount of concentrate offered. Our findings contrast with research by Zanine et al. (2019) who observed a longer grazing time and lower rumination time for cows offered a high (38.4 kg DM/d) PA compared with a medium (30.3 kg DM/d) or low (26.8 kg DM/d) PA. The number of graze bites was not different between cows offered different PA, which aligns with previous research at Ellinbank by Alvarez-Hess et al. (2021), who reported no effect of PA on the number of prehension bites. However, Werner et al. (2019) reported that the number of graze bites increased when PA was restricted to 60% of the normal allowance. In our experiment, the number of graze bites was greater for cows offered 4 kg DM of concentrate per day compared with those offered either 6 or 8 kg DM of concentrate per day in our experiment. This is most likely aligned with cows offered 4 kg DM of concentrate per day having an increased pasture DMI compared with cows offered 6 or 8 kg DM of concentrate per day, and therefore having an increased appetite for pasture when they returned to the paddock.

We acknowledge that the length of this experiment has its limitations. Although cows offered a PA of 50 or 65 kg DM/d had a greater BW compared with cows offered 20 kg DM/d (*P* = 0.003), the short length of this experiment may not have been sufficient for the full effects of PA on BW to be fully expressed. However, we do believe that the duration of this experiment was sufficient for effects on milk production and pasture intake to be observed. It is also important to note that high PA of 50 and 65 kg DM/cow per day, such as were offered to 6 groups of cows in this experiment, are unlikely to be offered on commercial dairy farms, regardless of the amount of concentrate offered. Pasture allowances of this magnitude were offered as an experimental treatment to assess potential pasture intake when pasture availability was not limiting, and to contribute to the additional aim of this experiment to validate DMI prediction equations under development (J. W. Heard, K. Giri, M. L. Douglas, and M. Wright, unpublished). Cows offered these high allowances underutilized the pasture, leading to high postgrazing pasture residuals, which would likely reduce pasture quality as the sward regrows. Additionally, the short duration of this experiment may be a limitation for the milk production and composition data reported, and longer-term follow-up experiments would be beneficial.

This experiment aimed to investigate different PA and concentrate amounts on milk production, individual cow pasture DMI, and grazing behavior. A combination of PA and concentrate amount will affect pasture utilization for farmers in temperate regions of the world with pasture-based systems, with flow-on effects for optimal pasture management and profitability. Increasing PA resulted in cows having an increased pasture DMI, but the consequence of this was higher postgrazing residuals and lower pasture utilization (decreasing from 54% for cows offered a PA of 20 kg DM/d to 27% for cows offered a PA of 65 kg DM/d). Therefore, the first hypothesis is accepted. Higher milk yields occurred when cows were offered PA at and above 35 kg DM/cow per day. Additionally, when cows were offered 6 or 8 kg DM of concentrate per day, milk yield was also increased; therefore, the second hypothesis is also accepted. Total daily eating time was not influenced by either PA, therefore the first part of the third hypothesis is rejected. Following on, as concentrate amount also did not influence eating time, the second part of the third hypothesis is accepted.

In summary, a PA between 20 and 35 kg DM/cow per day is appropriate for cows in early lactation, but considerations of the amount of concentrate offered are important to ensure good pasture utilization. Pasture allowances greater than 35 kg DM/cow per day result in inefficient use of the pasture and do not result in additional milk production, and are therefore not recommended. Additionally, cows offered 6 or 8 kg DM of concentrate per day had higher daily milk and milk protein yields compared with cows offered 4 kg DM concentrate per day, but pasture intake and therefore utilization was greater for cows offered the lowest amount of concentrate.
